# Effects of daphnetin on biofilm formation and motility of *pseudomonas aeruginosa*

**DOI:** 10.3389/fcimb.2022.1033540

**Published:** 2022-11-18

**Authors:** Zuoji Ye, Liumei Ye, Dingbin Li, Shunsheng Lin, Wusheng Deng, Li Zhang, Jinhua Liang, Jinlong Li, Qingjun Wei, Ke Wang

**Affiliations:** ^1^ Department of Orthopedic Trauma and Hand Surgery, The First Affiliated Hospital of Guangxi Medical University, Nanning, China; ^2^ Department of Pulmonary and Critical Care Medicine, The First Affiliated Hospital of Guangxi Medical University, Nanning, China; ^3^ Department of Dermatology and Venereology, The First Affiliated Hospital of Guangxi Medical University, Nanning, China

**Keywords:** biofilm, daphnetin, pseudomonas aeruginosa, motility, pyocyanin

## Abstract

**Introduction:**

*Pseudomonas aeruginosa* is a common clinical opportunistic pathogen. Antibiotic resistance of *P. aeruginosa* is frequent, and it affects the clinical curative effect and leads to recurrent infections, disease progression, and difficult treatment, especially in cystic fibrosis patients. The drug-resistance mechanism of *P. aeruginosa* is complex, and biofilms play an important role. Given the widespread antibiotic resistance of *P. aeruginosa*, the discovery of a drug that can prevent or eradicate biofilm formation is imperative. Daphnetin (DAP), a coumarin derivative, is a safe, non-toxic, natural compound with antibacterial and anti-biofilm properties. Herein, this study highlights the bacterial motility effects, antibacterial effect, pyocyanin production, and anti-biofilm potential of DAP against *P. aeruginosa*.

**Methods:**

In this study, the minimal inhibitory concentration of DAP against *P. aeruginosa* was determined using the microdilution method. The antibiofilm activity of DAP against *P. aeruginosa* was determined using crystal violet staining, colony-forming unit enumeration, and scanning electron microscopy. The effect of DAP on *P. aeruginosa* motility was detected using the swimming, swarming, and twitching agar plates to measure the diameter of the concentric area.

**Results:**

We found that DAP at concentrations of 0.445–1.781 mg/mL and 0.89–1.781 mg/mL can effectively inhibit biofilm formation and eradicate the formed biofilm of *P. aeruginosa*, respectively. DAP reduced pyocyanin production and inhibited bacterial motility of *P. aeruginosa*.

**Discussion:**

In conclusion, our results support the conclusion that DAP can effectively eradicate formed biofilm and inhibit biofilm formation, bacterial motility, and pyocyanin production of *P. aeruginosa* and may represent a natural anti-biofilm therapeutic agent.

## Introduction

*Pseudomonas aeruginosa* is a Gram-negative pathogen. It can cause hospital-acquired infections, infections in immunocompromised patients, and chronic infections in patients with cystic fibrosis (CF) (Reynolds and Kollef, 2021). It can also easily develop resistance to common antibiotics, which results in chronic recurrent infection (Azam and Khan, 2019). *P. aeruginosa* is also known to easily form biofilms after infection and produce a variety of virulence factors, such as pyocyanin, elastase, and protease, to evade the host’s immune system and effects of antibiotics (Paluch et al., 2020). The formation of biofilms is the main reason for refractory infection (Pang et al., 2019).

Biofilms are microbial aggregates encased in a self-produced extracellular polymer matrix that can be attached to the surface or free-floating (Townsley and Shank, 2017). However, with the increase in biofilm exploration, pathogenic biofilm matrices can be produced by other bacteria outside the biofilm (Bjarnsholt et al., 2013). Approximately 65% of infectious diseases and >80% of chronic infections are related to microbial biofilms (Cendra and Torrents, 2021). The extracellular polysaccharide matrix is mainly composed of alginate and carbohydrates that are secreted by bacteria. The penetration of the alginate layer by the originally effective antibiotics becomes difficult and drug desensitization results because of the protection offered by alginate (Moser et al., 2021). The current antibiotic treatment against *P. aeruginosa* is becoming ineffective because of the increasing drug resistance (Chatterjee et al., 2016). Hence, drugs or natural products with anti-biofilm and antibacterial activity that can increase the sensitivity of pathogens to antibiotics need to be discovered. An effective way to improve the antibacterial activity of drugs and increase the sensitivity of pathogens to antibiotics is to prevent the formation of a bacterial biofilm. Bacterial adhesion is the first step in biofilm formation, and swimming, swarming, and twitching motility play important roles during the early adhesion stage of bacterial biofilm formation (Rasamiravaka et al., 2015). Cell-cell interactions, the second step of biofilm formation, are regulated *via* the quorum sensing (QS) system, which includes a series of biological processes, such as the regulation of EPS matrix production, modulation of nutrient utilization, and expression of virulence factors (Guzman-Soto et al., 2021). Pyocyanin is one of the virulence factors secreted by *P. aeruginosa*, and it has been proven to promote biofilm formation and bacterial survival (Zhu et al., 2019). Biofilms can protect bacteria from antimicrobial treatment *via* several adaptive mechanisms (Maurice et al., 2018). Therefore, a new anti-biofilm strategy to disrupt the pyocyanin production and bacterial adhesion of *P. aeruginosa* is needed.

Nowadays, natural compounds have attracted increasing attention due to their remarkable anti-microbial effects and rare side effects (Rossiter et al., 2017; Qi et al., 2022). Natural drugs derived from Chinese herbs not only overcome resistance but also increase the susceptibility of pathogens to several non-natural drugs (Ye et al., 2020). Antibiotic overuse has led to extensive development of antibiotic resistance; therefore, it has become important to use non-antibiotic agents, such as safe natural compounds, to replace or complement traditional antibiotics for the control of pathogenic bacteria (Li et al., 2018).

Daphnetin (DAP), also 7,8-dihydroxycoumarin, is a traditional Chinese medicine (Zhang et al., 2020). Several studies have shown that it has anti-inflammatory (Zhang et al., 2019), anticancer (Mohamed et al., 2014), and neuroprotective effects (Huang et al., 2006). DAP has been used to treat rheumatoid arthritis and coagulopathy in clinic (Wu et al., 2019). Furthermore, DAP had been shown antibacterial activity and anti-biofilm effect of *Ralstonia solanacearum* (Yang et al., 2016; Yang et al., 2017). In summary, DAP is a traditional Chinese medicine with various effects. However, to our knowledge, no research has yet been conducted on the biofilm inhibition and eradication effects of DAP against *P. aeruginosa*. In this experiment, DAP was used against *P. aeruginosa* PAO1 to observe its effects on bacterial growth, biofilm formation, motility, and pyocyanin production to assess its potential as a new drug for the clinical treatment of drug-resistant infections of *P. aeruginosa* and new strategies for the study of the mechanism underlying biofilm formation.

## Materials and methods

### Bacterial culture and reagents

The *P. aeruginosa* PAO1 wild-type strain was donated by the Singapore Centre on Environmental Life Sciences Engineering, Nanyang Technological University, Singapore (Chua et al., 2013). Before each experiment, the PAO1 wild-type strain was smeared in Luria-Bertani (LB) agar plate (LB Agar, Beijing Land Bridge Technology Co., Ltd., Guangzhou, China) and cultured in a 37°C incubator (HPX-400, Shanghai Yuejin Medical Equipment Co., Ltd., Shanghai, China) for 24 h. A single colony with good growth was selected and transferred to a 3-mL LB culture medium (Guangdong Huankai Microbial Technology Co., Ltd., Guangzhou, China) and incubated in a shaker (THZ-82, Changzhou Zhibo Instrument Manufacturing Co., Ltd., Changzhou, China) at 37°C and 220 rpm for 16 h to obtain a bacterial suspension. An appropriate volume of the overnight bacterial suspension was diluted to an optical density (OD) value at 600 nm (OD_600_) of 0.1 (ultraviolet [UV]-Visible Spectrophotometer T6, Beijing Purse General Instrument Co., Ltd., Beijing, China) in LB broth for subsequent experiments. DAP (purity >97%) was purchased from Shanghai Macklin Biochemical Co., Ltd. (Shanghai, China) and DAP (178.1 mg) was dissolved in 1mL dimethyl sulfoxide (Beijing Solarbio Science Technology Co., Ltd., Guangzhou, China) to yield a DAP stock solution. Four experimental groups were established: (a) DAP group, (b) drug solvent control group, (c) PAO1 group (untreated group), and (d) sterile medium group. All experiments were repeated in triplicate.

### Determination of minimum inhibitory concentration

The MIC of DAP against *P. aeruginosa* was determined using the Nature protocol (Belanger and Hancock, 2021). The DAP stock solution was diluted to 3.562 mg/mL with Müller–Hinton broth (Qingdao Hopebio Biotechnology Co., Ltd., Qingdao, China), and the dilution of the dimethyl sulfoxide (DMSO) was 100× at the same time. To each well of a 96-well plate, 95 µL of Müller–Hinton broth (MHB) was added. DAP (95 µL) at 3.562 mg/mL was added to the first lane of the plate containing 95 µL of MHB and mixed well (1:2 dilution). Subsequently, 95 µL of the mixture was transferred to the next well. To the sixth lane, 95 µL solution was discarded to form DAP dilution concentrations with each well containing a final volume of 95 µL. The final tested drug concentrations ranged from 0.055 to 1.781 mg/mL, leaving three lanes for growth, negative sterility, and drug solvent controls. Finally, 5 µL (OD_600_ = 0.01) of the bacterial solution was added to the test well and growth control group to yield a bacterial concentration of 5 × 10^5^ colony-forming units (CFUs)/mL. The drug solvent control was added to 5 µL bacterial solution and DMSO (the final dilution of the DMSO was 100×). The 96-well assay plates were cultured statically at 37 °C for 18 h, after which time the MIC was determined based on the turbidity of the culture, and the optical density was recorded at 600 nm (OD_600_) to quantify the overall bacterial growth in each well.

### Method for detecting DAP-induced effects on the growth of *P. aeruginosa*

The method for detecting DAP-induced growth was based on reports by previous studies (Luo et al., 2016) with some modifications. A bacterial solution with an OD_600_ of 0.1 was obtained according to the above method. For the drug groups, bacterial suspensions were mixed with a series of different concentrations of DAP (0.055–1.781 mg/mL). The bacterial suspension and sterile LB medium were added to the growth control and negative sterility control, respectively. The solvent control was added to the bacterial solution and DMSO and diluted 100 times. The solution for each group was fully mixed and added to a 96-well plate, 200 µL per well, with six wells repeated for each group. The 96-well plate was cultured at 37°C for 24 h on a shaker incubator (220 rpm). Turbidity was measured every 3 h at OD_600_, and growth curves were recorded.

### Methods for detecting the antibiofilm activity of DAP against *P. aeruginosa*

#### Biofilm inhibition assay

The inhibitory effects of DAP against *P. aeruginosa* PAO1 biofilm formation were determined using a protocol from Nature’s method (Haney et al., 2021) with some modifications. Briefly, a bacterial solution with OD_600_ of 0.1 (approximately 1×10^8^ CFU/mL) was obtained. The growth control, negative sterility control, and solvent control were obtained by the method described above. For the DAP groups, different concentrations (0.055–1.781 mg/mL) of DAP were mixed with bacterial suspensions. The solution in each group was thoroughly mixed and added to a 96-well plate at 200 µL per well, and six wells were repeated for each group. The 96-well plate was cultured at 37 °C for 20 h. Bacterial growth results were collected before crystal violet staining, and the OD_600_ of the 96-well plate was recorded to quantify overall bacterial growth in each well. After that step, non-adherent cells in the supernatants were removed by pipetting, and the plate was cleaned gently with 200 µL sterile phosphate-buffered saline (Beijing Solarbio Science Technology Co., Ltd., Beijing, China) three times. After drying at room temperature, the biofilm biomass was assessed using a crystal Violet assay. Each well was stained with 210 µL 0.1% (w/v) crystal violet staining solution (100 mL, Beijing Solarbio Science & Technology Co., Ltd., Beijing, China) at room temperature for 30 min. The crystal violet staining solution was removed from all wells, and each well was rinsed three times with distilled water. The dissolved crystal violet was resuspended in 220 µL 70% ethanol (100 mL, Shanghai Jizhi Biochemical Technology Co., Ltd., Shanghai, China) after drying and incubated for 30 min at room temperature with gentle shaking. Its absorbance value was measured at OD_595_.

#### Biofilm eradication assay

The method for evaluating the *P. aeruginosa* biofilm eradication effects of DAP was based on the literature (Haney et al., 2021). A bacterial solution with an OD_600_ of 0.1 (approximately 1×10^8^ CFU/mL) was obtained. Two hundred microliters of a bacterial suspension with OD_600_ of 0.1 was added to the interior well of the microtiter plate to establish biofilms in 96-well plates, and the remaining wells received 200 µL sterile LB medium. Two identical 96-well microtiter plates were labeled. One plate was used for crystal violet staining, and the other one was used for 2, 3, 5-triphenyl-2H-tetrazolium chloride staining. The plates were incubated at 37°C for 24 h under static conditions. Subsequently, the OD_600_ of the bacterial growth within the wells before treatment was recorded. The supernatants containing non-adherent cells were removed after that, and biofilms were gently rinsed three times with sterile phosphate-buffered saline (PBS). Existing biofilms were incubated at 37°C in LB supplemented with the test agents (a series of different concentrations of DAP ranging from 0.055 to 1.781 mg/mL) for 20 h. The biofilm biomass was assessed as described above. To the TTC plate, 5 µL of a 2% (w/v) sterile-filtered solution of TTC (100 mL, Beijing Solarbio Science & Technology Co., Ltd., Beijing, China) was added, and the plate was incubated for 20 h at 37°C under static conditions. The spent growth media in the TTC plate was discarded by gently inverting the plates and rinsing all the wells of the TTC plate three times with distilled water. After drying at room temperature, the TTC dye was removed from the TTC plate by adding 205 µL of DMSO to each well and incubating for 30 min at room temperature with gentle shaking. The absorbance at 500 nm was recorded for the TTC plate. Six wells were repeated per group.

#### CFU enumeration assay

Viable bacteria in the biofilm were evaluated by obtaining colony counts according to a previously described method (He et al., 2014; Luo et al., 2017) and recording the OD_600_ for bacterial growth in the desired experimental wells. The medium was gently discarded from each well of the 96-well plate after culturing for 24 h and washed three times with sterile PBS. Next, 200 µL of 0.1% (v/v) Triton X-100 (100 mL, Shanghai Biyuntian Biotechnology Co., Ltd., Shanghai, China) was added to each well and vibrated with an ultrasound (JP-031S, Shenzhen Jiemeng Cleaning Equipment Co., Ltd., Shenzhen, China) for 5 min. Ten-fold serial dilutions of the bacterial cell suspensions from the biofilm cells were prepared, and serial dilutions from each well were plated onto LB agar plates for enumeration and counting, after which the colony number and its corresponding dilution were recorded. The count was repeated for three wells per group.

### Biofilm imaging under a scanning electron microscope

Morphological changes (inhibitory effects and eradication effects) in biofilms after DAP treatment were analyzed using a scanning electron microscope based on an approach reported by a previous study (Luo et al., 2017). A polyvinyl chloride flexible film was cut into a circle with a 1-cm diameter and soaked in 75% alcohol overnight under ultraviolet irradiation, after which the alcohol on the surface was washed with sterile 0.9% normal saline to obtain the biofilm carrier. The bacterial solution with bacterial concentration OD_600_ of 0.1 (approximately 1×10 8 CFU/mL) was obtained according to the above-mentioned method. To test the inhibitory effect of DAP, one prepared carrier was inserted into each well in the 24-well plate, completely immersed in the bacterial solution (in the presence or absence of tested drug concentrations ranging from 0.222 to 1.781 mg/mL), and cultured at 37°C for 24 h. To examine the biofilm eradication effects of DAP, biofilm models were established and treated with different concentrations of DAP (ranging from 0.222 to 1.781 mg/mL) at 37°C for 24 h. The biofilm carriers were collected, and the planktonic bacteria on the carrier surface were gently cleaned three times using sterile PBS. Next, specimens were fixed with a 3% (w/v) glutaraldehyde solution (Wuhan Saiweier Biotechnology Co., Ltd., Wuhan, China) at 4°C for 2 h. The carriers were once again cleaned three times using sterilized PBS. The glutaraldehyde on the carrier surface was washed (10 min/wash), followed by dehydration in a graded series of ethanol (30%, 50%, 70%, 80%, and 90%) at different concentrations for 10 min/wash. The carriers were dehydrated three times in 100% ethanol (10 min/wash) after this. After drying, gold powder was sprayed under a vacuum, and the biofilms were observed under scanning electron microscopy.

### Methods for detecting DAP effects on *P. aeruginosa* motility

#### Swimming motility detection

The PAO1 strain was seeded on LB agar and cultured at 37°C for 24 h, after which one colony was inoculated in the presence or absence (control) of tested drug concentrations ranging from 0.222 to 1.781 mg/mL on the surface of swimming agar plates containing 1.0% (w/v) tryptone (OXOID, LP0042), 0.5% (w/v) sodium chloride (Chengdu Jinshan Chemical Reagent Co., Ltd., Chengdu, China), and 0.3% (w/v) agar (SIGMA, A1296, Switzerland). The diameter of the concentric area was centered on the inoculation point (Saeki et al., 2021). Three plates were used for each group.

#### Swarming motility detection

*P. aeruginosa* was grown in LB broth for 24 h at 37°C. Swarming agar plates with 1% (w/v) glucose (Tianjin Kemio Chemical Reagent Co., Ltd., Tianjin, China), 0.5% (w/v) peptone (Qingdao Hope Bio-Technology Co., Ltd., Qingdao, China), 0.2% (w/v) yeast extract (SIGMA, Y1625, Switzerland), 0.5% (w/v) agar, and different concentrations of DAP were prepared for use. Untreated plates did not contain DAP. Ten microliters of a *P. aeruginosa* suspension were used to inoculate the center of the swarming agar plates and cultured at 37°C for 24 h. The shape of the area centered on the bacterial inoculation point was observed, and the diameter was measured (Saeki et al., 2021). Three plates were used for each group.

#### Twitching motility detection

The PAO1 strain was seeded on LB agar plates and incubated at 37°C for 24 h. The twitching agar plate composition consisted of several ingredients: (a) 1.0% (w/v) tryptone, (b) 0.5% (w/v) yeast extract, (c) 1.0% (w/v) sodium chloride, and (d) 1.0% (w/v) agar in the presence and absence (control) of tested drug concentrations ranging from 0.222 to 1.781 mg/mL. A single colony cultured overnight was selected with a sterile toothpick and inoculated at the bottom on the center of the twitching agar plates. Plates were cultured upside down at 37°C for 24 h, and the shape of the colony that formed with the inoculation point as the center was observed. The agar in the plates was gently selected with a toothpick, and non-adherent bacteria at the bottom of the plates were washed with sterile PBS and stained with 1% crystal violet solution for 20 min. The unbound crystal violet was rinsed with sterile PBS, and the diameter of the area centered on the bacterial inoculation point was measured (Saeki et al., 2021). Three plates were used for each group.

### Pyocyanin estimation

Pyocyanin production was measured by performing a quantitative chemical assay based on methods described in previous studies (Luo et al., 2017; Zhu et al., 2019). Overnight cultures of *P. aeruginosa* PAO1 were adjusted to an OD_600_ of 1.0 in LB medium and incubated with tested drug (DAP) concentrations ranging from 0.222 to 1.781mg/mL for 24 h. The control group contained just *P. aeruginosa* PAO1 without agents. After treatment, each group of the culture samples was collected and centrifuged at 10,000 g for 15 min. The supernatant was passed through a 0.22-μM filter (Biosharp, Beijing Labgic Technology Co., Ltd. Beijing, China). Three milliliters of the culture supernatant were extracted with chloroform at a ratio of 3:2, followed by extraction with 1 mL of 0.2 M HCl. The supernatant was transferred to a 96-well plate, and the pyocyanin in the supernatant was quantified at OD_520_. Five wells were used for each group.

### Statistical analysis

Data were analyzed with the GraphPad Prism software version 8.0 using descriptive (mean ± standard deviation [SD]) statistics. One-way analysis of variance (ANOVA) was used for multiple group comparisons, and the comparisons were pairwise. *P*-values of <0.05 denoted statistical significance.

## Results

### MICs

**Figure 1:**
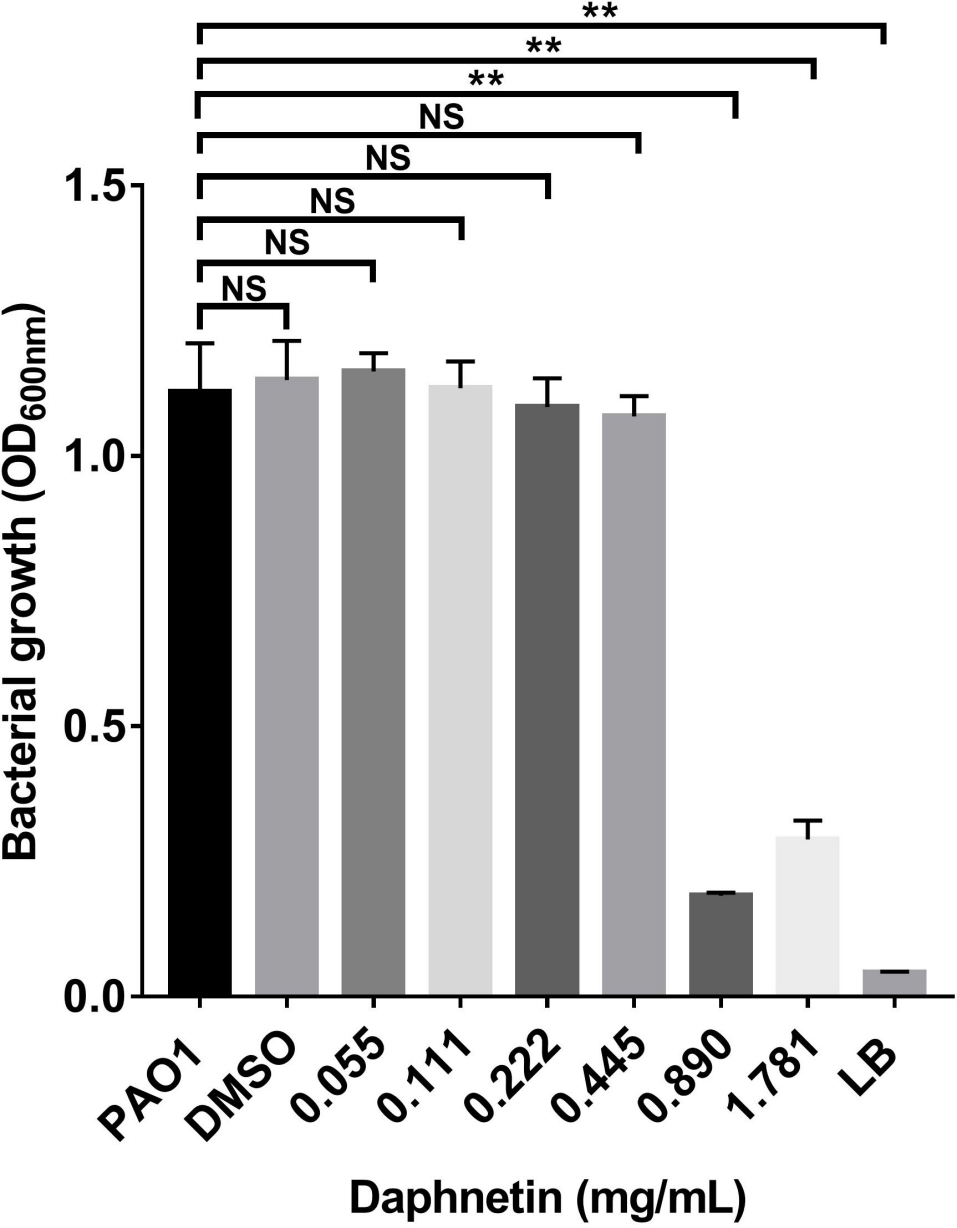
The minimal inhibitory concentration (MIC) of daphnetin (DAP) against planktonic *Pseudomonas aeruginosa* PAO1. The bacterial growth of *Pseudomonas aeruginosa* treated with varying concentrations of DAP (0.055–1.781 mg/mL) was evaluated using optical density (OD_600_ nm). Experiments were performed in triplicate; NS indicates P-values of >0.05 and ** indicate P-values <0.01, relative to the PAO1 control (no DAP).

The MIC of the test drug was determined using the bacterial growth based on the turbidity and the optical density at 600 nm of the culture. As shown in **Table 1** and **Figure 1**, the MIC of DAP against *P. aeruginosa* was 0.890 mg/mL. The PAO1 and DAP groups with concentrations below 0.89 mg/ml were turbid, and the sterile LB and DAP groups with concentrations of 0.890 and 1.781 mg/ml, respectively, were clear. The OD_600_ of each group was also consistent with the DAP MIC of 0.890 mg/mL. Indicating DAP exerted no direct bactericidal effect and bacteriostatic effect on planktonic P. aeruginosa PAO1 cells when DAP concentration below 0.890 mg/mL.

**Table 1:** Susceptibility of *P. aeruginosa* PAO1 to daphnetin (DAP).

Concentration of DAP	Turbidity
PAO1 control	**+++**
DMSO (solvent control)	**+++**
0.055 (mg/mL)	**+++**
0.111 (mg/mL)	**+++**
0.222 (mg/mL)	**+++**
0.445 (mg/mL)	**+++**
0.89 (mg/mL)	**–**
1.781 (mg/mL)	**–**
LB control	**–**

**+++** and **–** represent obvious turbidity and clear, respectively.

### Effect of DAP on the growthof *P. aeruginosa*

**Figure 2:**
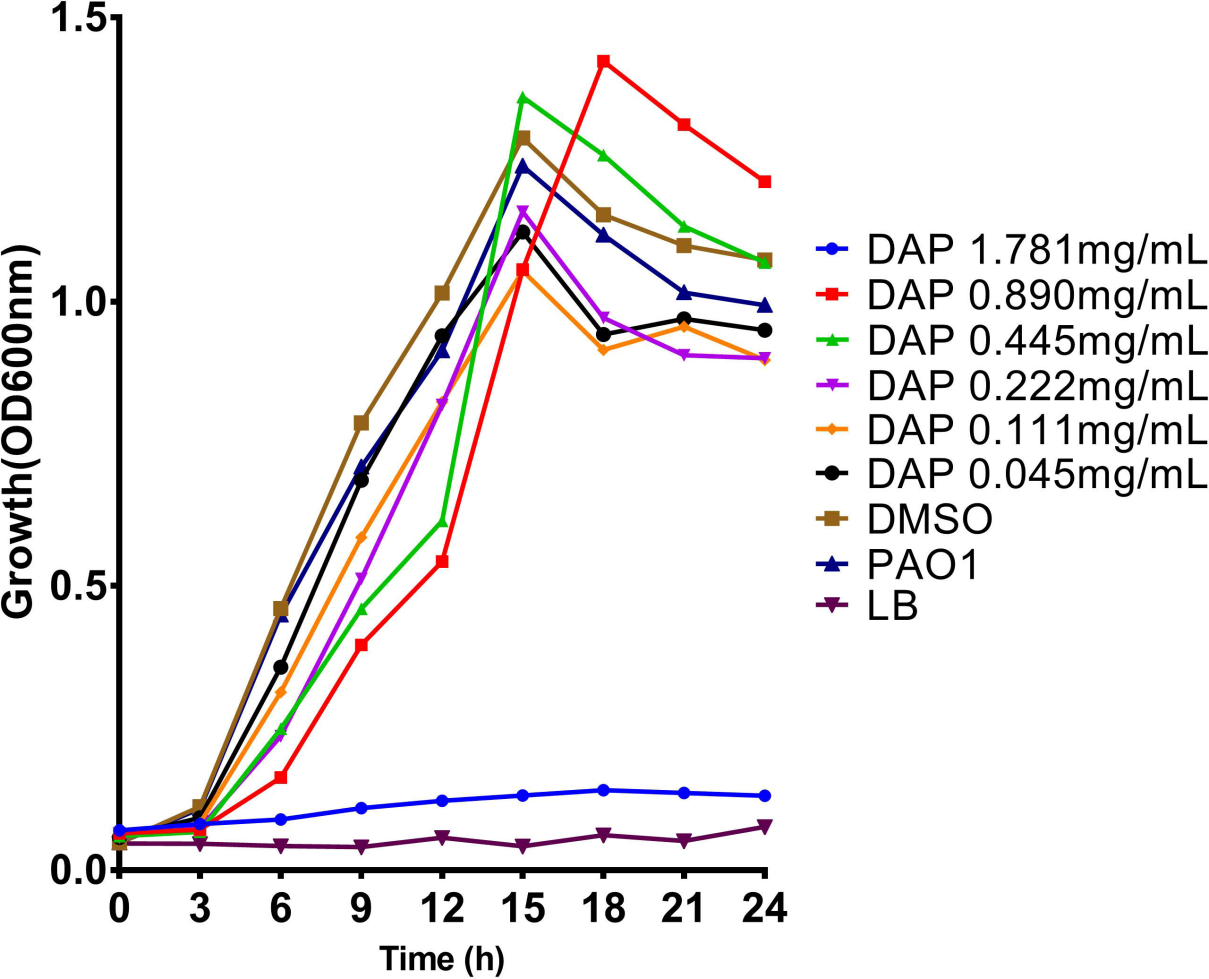
Effect of daphnetin (DAP) on the growth of *P. aeruginosa* within 24 h. Growth curves of *P. aeruginosa* PAO1 treated with different concentrations of DAP (0.055–1.781 mg/mL) in LB broth at 37°C for 24 h. The error bars represent the standard error of the OD_600_ value for each time point on the growth curves.

To study the effects of DAP on biofilm formation, motility, and pyocyanin production of *P. aeruginosa*, the experiment was carried out at sub-inhibitory concentrations. DAP at concentrations ≤ 0.890 mg/ml and the drug solvent (DMSO) did not affect bacterial growth relative to the PAO1 control group (*P* > 0.05) (**Figure 2**), indicating that DAP had almost no significant effect on the growth of *P. aeruginosa* at sub-inhibitory concentrations. However, the cell density and kinetic growth of planktonic *P. aeruginosa* PAO1 were inhibited at the concentration of twice the MIC (1.781 mg/mL) (*P* < 0.05).

### Inhibitory effect of DAP on biofilm formation against *P. aeruginosa*

**Figure 3:**
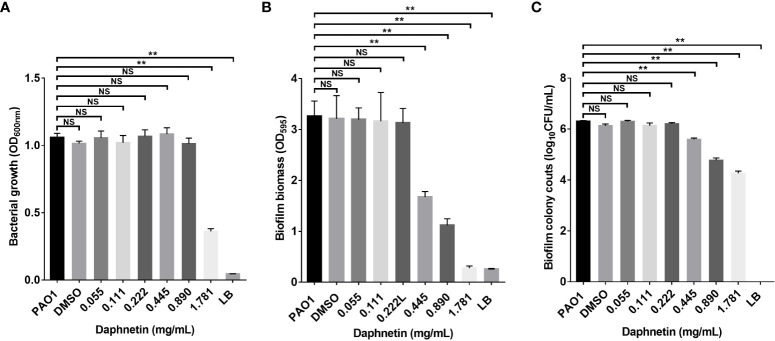
The biofilm inhibition effect of daphnetin (DAP) against *P. aeruginosa*. Bacterial suspensions were seeded in 96-well plates exposed to different concentrations of DAP (0.055, 0.111, 0.222, 0.445, 0.890, and 1.781 mg/mL) for 20 h, and biofilm mass formation and bacterial counts were quantified in triplicate. **(A)** Planktonic bacterial growth evaluation. **(B)** Crystal violet staining. **(C)** Biofilm bacterial counts. Results are represented as the mean ± standard deviation (SD). NS indicates P-values >0.05 and ** indicate P-values <0.01.

After the suspension of *P. aeruginosa* was cultured with different concentrations of DAP for 24 h, biofilms were formed on the inner wall of the 96-well plate. Bacterial growth in each well in the 96-well plate was first evaluated, and DAP inhibited bacterial growth at a concentration of 1.781 mg/mL (P < 0.05), while other concentrations of DAP and DMSO did not affect the bacterial growth of *P. aeruginosa* (**Figure 3A**). The crystal violet staining results reflected the amount of biofilm formation. As shown in **Figure 3B**, the amount of biofilm formation gradually decreased at the effective concentrations (ranging from 0.445 to 1.781 mg/mL) and the difference was statistically significant when compared with PAO1 group (P < 0.05). Indicating that DAP can inhibit the formation of *P. aeruginosa* biofilms, and the inhibitory effect was gradually enhanced with the increase in concentration. When the DAP concentration was < 0.445 mg/mL, DAP showed no inhibitory effect on biofilm formation. The drug solvent (DMSO diluted 100 times) also did not affect PAO1 biofilm formation, and no biofilm formation was found in the LB group. DAP Concentrations of 0.445, 0.89, and 1.781 mg/mL had effects on the biofilm bacterial counts and the difference was significant when compared with PAO1 group (P < 0.05), and the number of bacteria in the biofilm gradually decreased with the increase in concentration. However, concentrations of < 0.445 mg/mL had no obvious effects on biofilm bacterial counts and the difference was not statistically significant (P > 0.05). The biofilm bacterial counts in all groups are shown in **Figure 3C**.

### Eradication of *P. aeruginosa* biofilm by DAP

**Figure 4:**
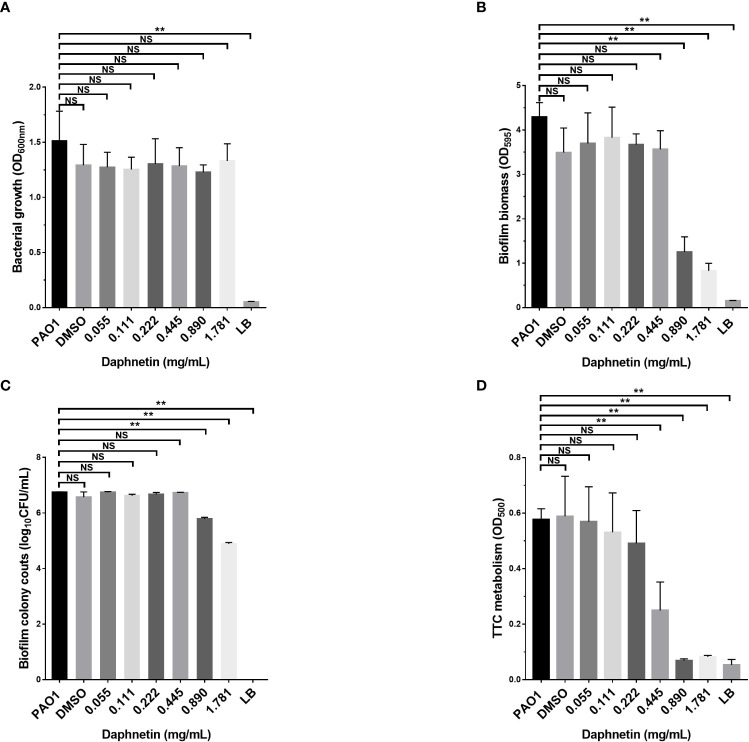
The biofilm eradication effect of daphnetin (DAP) against *P. aeruginosa*. Biofilms of *P. aeruginosa* were formed in 96-well plates after 24 h culture, and the pre-formed biofilms were treated with different concentrations of DAP (0.055, 0.111, 0.222, 0.445, 0.890, and 1.781 mg/mL) for 20 h. The biofilm mass, bacterial counts, bacterial metabolism, and biofilm biomass before treatment were quantified in triplicate. **(A)** Biofilm formation before treatment. **(B)** Crystal violet staining. **(C)** Biofilm bacterial counts. **(D)** TTC staining. Results are represented as the mean ± SD; NS indicates P-values >0.05 and ** indicate P-values <0.01.

The *P. aeruginosa* biofilm eradication effects of DAP were further investigated. The assessment of biofilm biomass in each group before treatment is shown in **Figure 4A**; the amount of biofilm was equal in each group. The remaining biofilm biomass was assessed after the formed biofilm was treated with different concentrations of DAP for 24 h. The results of crystal violet staining and biofilm bacterial counts in all groups are shown in **Figures 4B, C**. The amount of biofilm biomass gradually decreased at the effective concentration (0.89 and 1.781 mg/mL) and the difference was significant when compared with PAO1 group (P < 0.05), indicating that DAP could effectively eradicate *P. aeruginosa* biofilms. DAP did not affect the colony counts or biomass of *P. aeruginosa* PAO1 biofilms when the concentration was < 0.89 mg/mL. The drug solvent (DMSO diluted 100 times) also produced no effects on the PAO1 biofilm. The results of TTC staining are shown in **Figure 4D**. DAP inhibited the bacterial metabolism of *P. aeruginosa* at concentrations ranging from 0.445 to 1.781 mg/mL (P < 0.05).

### Effect of DAP against biofilm by scanning electron microscopy

**Figure 5:**
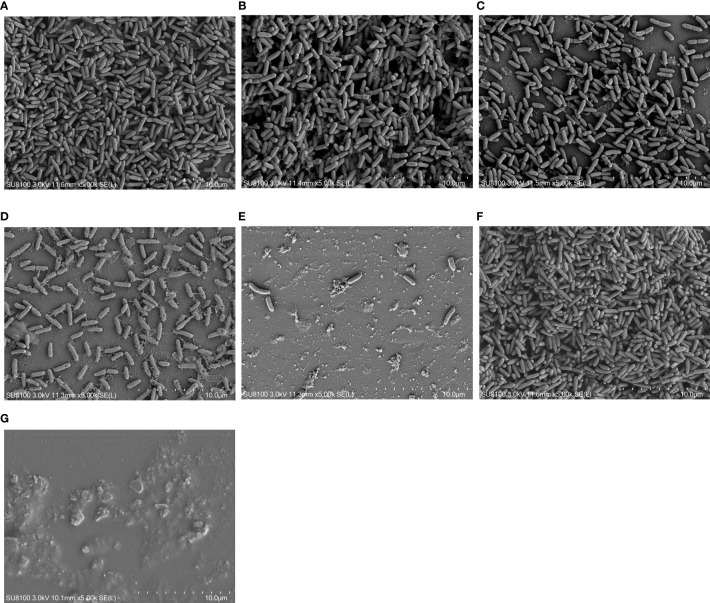
Inhibitory effect of daphnetin (DAP) on biofilm formation of *P. aeruginosa* is observed by electron microscopy after 24 h in culture (×5000). **(A)** PAO1 group. A large number of aggregated *P. aeruginosa* and their secreted matrices form the biofilm. **(B)** DAP group with final concentration of 0.222 (mg/mL). **(C)** DAP group with final concentration of 0.445 (mg/mL). **(D)** DAP group with final concentration of 0.890 (mg/mL). **(E)** DAP group with final concentration of 1.781 (mg/mL). In the DAP groups, bacterial aggregation decreased with increasing concentration, and a few shriveled bacteria can be observed. **(F)** Dimethyl sulfoxide (DMSO) group, drug solvent negative control group (no DAP), similar to the PAO1 group. **(G)** LB group, culture medium negative control group (no bacteria). DAP, Daphnetin; DMSO, dimethyl sulfoxide; LB, Luria-Bertani.

**Figure 6:**
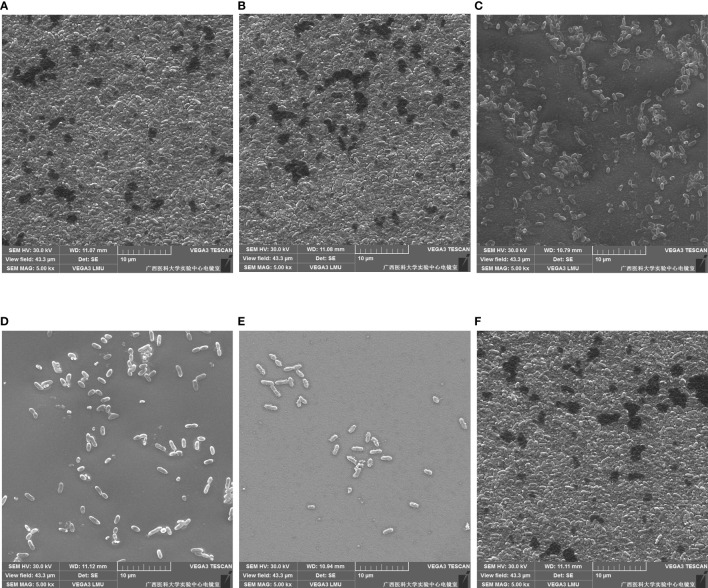
Eradication effect of daphnetin (DAP) against biofilm of *P. aeruginosa* is observed by electron microscopy after 24 h in culture (×5000). **(A)** PAO1 group. A large number of aggregated P. aeruginosa form the biofilm. **(B)** DAP group with final concentration of 0.222 (mg/mL). **(C)** DAP group with final concentration of 0.445 (mg/mL). **(D)** DAP group with final concentration of 0.890 (mg/mL). **(E)** DAP group with final concentration of 1.781 (mg/mL). In the DAP groups, bacterial aggregation decreased with increasing concentration. **(F)** Dimethyl sulfoxide (DMSO) group, drug solvent negative control group (no DAP).

The inhibitory and eradication effects of different concentrations of DAP against *P. aeruginosa* biofilms were investigated *via* SEM. Biofilm inhibition electron microscopy showed that *P. aeruginosa* grew extensively to form an early biofilm after 24 h of culture (**Figure 5A**). In the DAP groups, the bacterial density became sparse with increasing DAP concentration, and the size of some bacterial colonies shrank because of the effect of DAP (**Figures 5B–E**). DMSO diluted 100 times had no effect on biofilm formation (**Figure 5F**), and no biofilm formation in the LB group was detected (**Figure 5G**). Biofilm eradication electron microscopy showed that the number of P. aeruginosa PAO1 cells decreased with the increase in DAP concentration, and the existing cells showed a scattered distribution (**Figure 6**).

### Effect of DAP on *P. aeruginosa* motility

#### Swimming motility results

**Figure 7:**
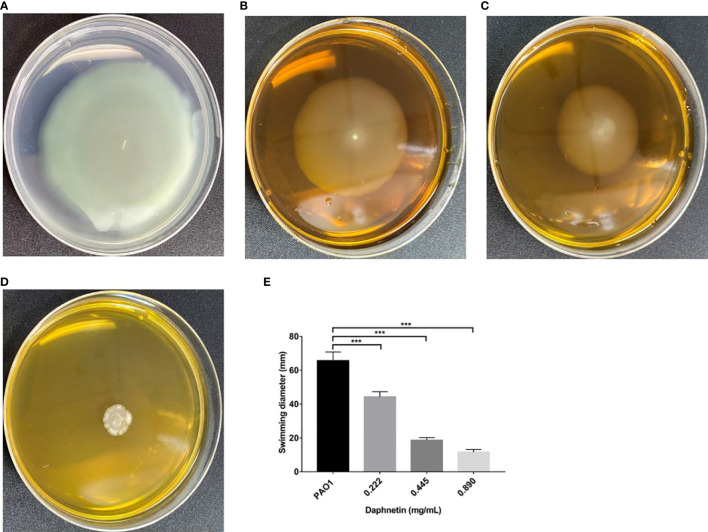
Effect of daphnetin (DAP) on swimming motility of *P. aeruginosa*. Swimming motility assay was conducted on plates containing different concentrations of DAP and some without DAP (Untreated Control). **(A)** PAO1 group. **(B)** DAP group with final concentration of 0.222 (mg/mL). **(C)** DAP group with final concentration of 0.445 (mg/mL). **(D)** DAP group with final concentration of 0.890 (mg/mL). **(E)** Swimming diameter of each group. Results are represented as the mean ± SD; *** indicate P-values <0.001. Analysis of variance (ANOVA) test was used to compare the groups (600×600 DPI).

As shown in **Figure 7**, a cloud-like concentric circle was formed with the inoculation point as the center in the PAO1 group, and the diameter was 70.67 ± 4.04 cm. The diameters in the 0.890, 0.445, and 0.222 mg/mL DAP groups were 13.67 ± 1.53, 23.00 ± 1.00, and 37.00 ± 1.00 mm, respectively. The difference was significantly less than that in the PAO1 group (P < 0.001).

#### Twitching motility results

**Figure 8:**
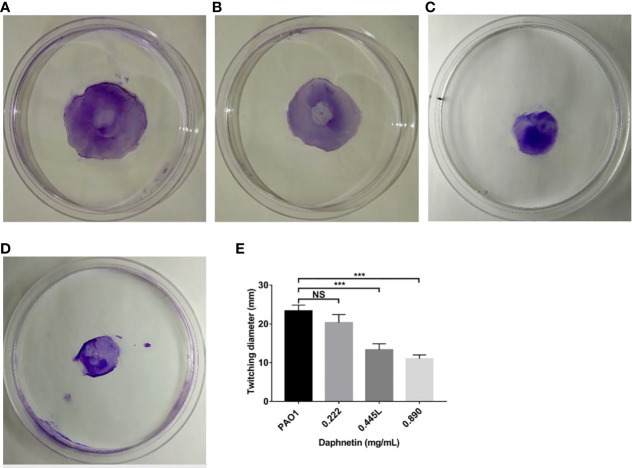
Effect of daphnetin (DAP) on twitching motility of *P. aeruginosa*. Twitching motility assay was conducted on plates containing different concentrations of DAP and in the absence of DAP (Untreated Control). **(A)** PAO1 group. **(B)** DAP group with final concentration of 0.222 (mg/mL). **(C)** DAP group with final concentration of 0.445 (mg/mL). **(D)** DAP group with final concentration of 0.890 (mg/mL). **(E)** Twitching diameter of each group. Results are represented as the mean ± SD; NS indicates P-values >0.05 and *** indicate P-values <0.001. ANOVA test was used to compare the groups (600×600 DPI).

The PAO1 group formed a disc-like structure at the inoculation point at the center, and its periphery showed gear-like radial pseudopodia (**Figure 8A**). The DAP group at the concentration of 0.222 mg/mL, as with the PAO1 group, formed a disc-like structure and pseudopodia (**Figure 8B**). The DAP groups (0.445 and 0.89 mg/mL) also formed a disc-like structure centered on the inoculation point, but the periphery was smooth without radioactive pseudopodia (**Figure 8C, D**). The diameters of each group were shown in **Figure 8E**, the DAP groups of 0.89 mg/mL (11.00 ± 1.00 mm) and 0.445 mg/mL (13.33 ± 1.53 mm) were smaller than those of the PAO1 group (23.33 ± 1.53 mm), and the difference was statistically significant (P < 0.001). The diameter of the 0.222 mg/mL DAP group (20.33 ± 2.08 mm) was almost the same as that in the PAO1 group, and the difference was not statistically significant (P > 0.05).

#### Swarming motility results

**Figure 9:**
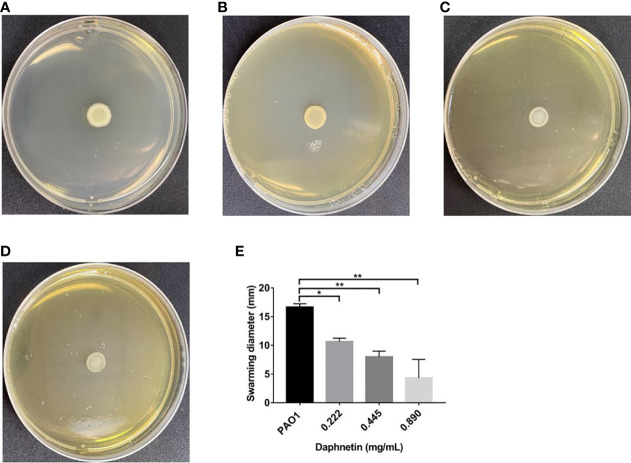
Effect of daphnetin (DAP) on swarming motility of *P. aeruginosa*. Swarming motility assay was conducted on plates containing different concentrations of DAP and in the absence of DAP (Untreated Control). **(A)** PAO1 group. **(B)** DAP group with final concentration of 0.222 (mg/mL). **(C)** DAP group with final concentration of 0.445 (mg/mL). **(D)** DAP group with final concentration of 0.890 (mg/mL). **(E)** Swarming diameter of each group. Results are represented as the mean ± SD; ** and * indicate P-values < 0.01 and < 0.05, respectively. ANOVA was used to compare the groups (600×600 DPI).

In the PAO1 group, concentric circular areas were formed around the inoculation point. As shown in **Figure 9**, the diameter of the 0.222 mg/mL DAP group (10.67 ± 0.58 mm) was shorter than that of the PAO1 group (16.67 ± 0.58 mm), and the difference was statistically significant (P < 0.05). The diameters of the 0.89 mg/mL (4.33 ± 3.213 mm) and 0.445 mg/mL (8.00 ± 1.00 mm) DAP groups were shorter than that of the PAO1 group; the differences were statistically significant (P < 0.01).

### Effect of DAP on pyocyanin production of *P. aeruginosa*

**Figure 10:**
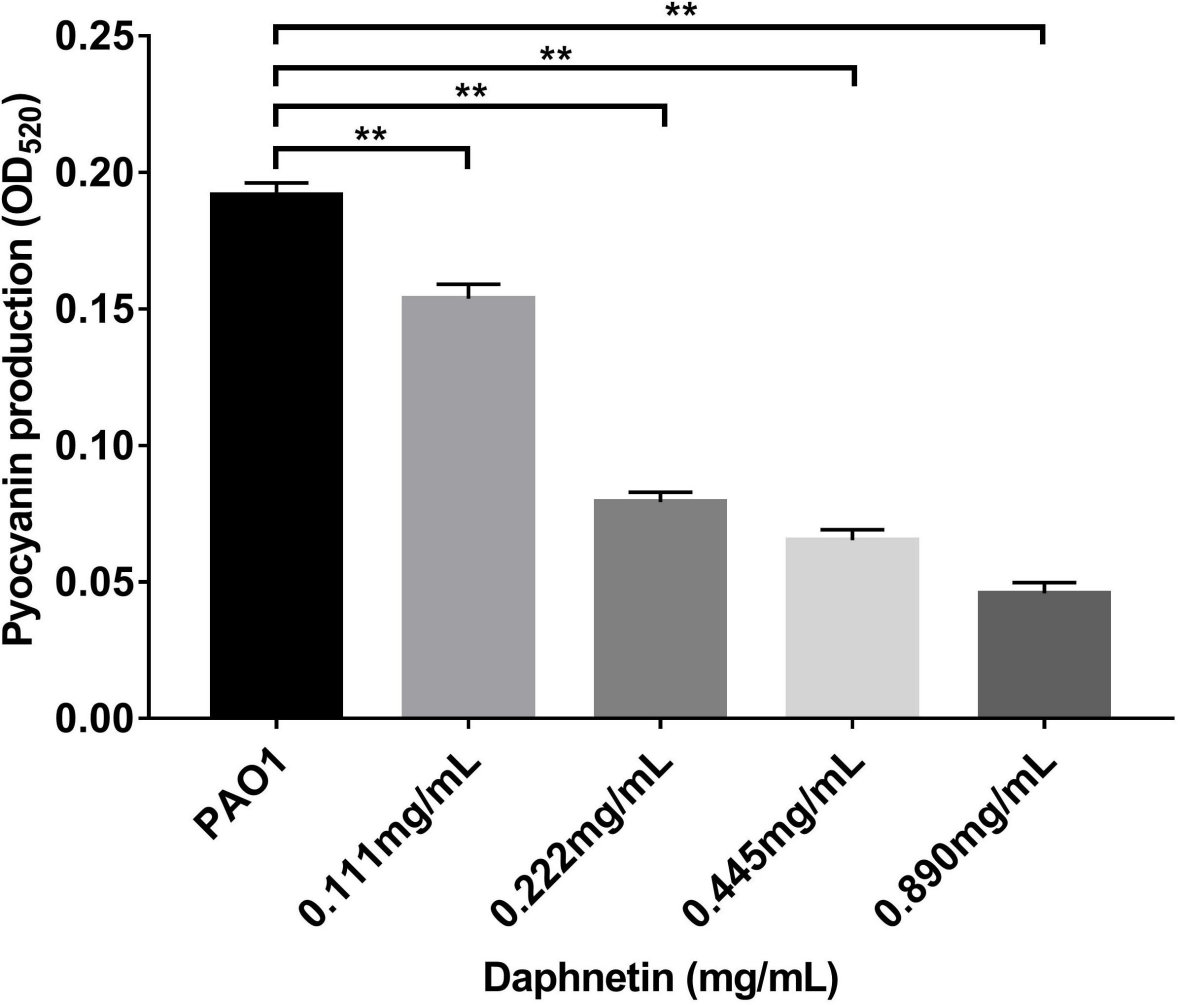
Effects of daphnetin (DAP) on pyocyanin production by *P. aeruginosa*. Pyocyanin production was measured at OD_520_ by a quantitative chemical assay. Error bars indicate the standard deviations. Results are represented as the mean ± SD; ** indicate P-values <0.01. ANOVA was used to compare the groups (600×600 DPI).

The effects of DAP on the pyocyanin production of *P. aeruginosa* were further examined (**Figure 10**). The pyocyanin production was the highest in the PAO1 group. With an increase in DAP concentration (ranging from 0.111 to 0.890 mg/mL), pyocyanin production significantly decreased (P < 0.05). Therefore, DAP inhibited the pyocyanin production of *P. aeruginosa*, and the inhibitory effect increased in a dose-dependent manner.

## Discussion

*P. aeruginosa* is a typical biofilm-forming microbe. It can secret virulence factors and develop drug resistance through the coordination of gene expression and QS systems (Wu et al., 2014). The QS system is responsible for biofilm formation, drug resistance, virulence factor expression, and motility of *P. aeruginosa* (Yin et al., 2015); therefore, the development of biofilm inhibiting agents can be used as a new strategy for antibacterial and antibiofilm treatment. Recent studies have shown that natural agents having plant secondary metabolites can disrupt biofilms (Lahiri et al., 2019), some compounds acquired from plants can be used for anti-biofilm (Luo et al., 2017). Daphnetin (DAP), a plant extract (Yang et al., 2016), has been found to exhibit antibacterial activity and anti-biofilm effects and cause a significant reduction in the virulence of *Ralsonia solanacearum* (Yang et al., 2021). However, the antibacterial and anti-biofilm effects of DAP against *P. aeruginosa* have not been previously studied. In this study, it was demonstrated that DAP could inhibit the biofilm formation and bacterial motility of *P. aeruginosa* for the first time. Our results and morphological observations (SEM) also indicated that DAP can effectively eradicate the formed biofilm of *P. aeruginosa*. Similar to the results of a previous literature study that reported that DAP reduced the virulence of *R. solanacearum* (Yang et al., 2021), our study demonstrated that DAP inhibited the production of *P. aeruginosa* pyocyanin. Based on these findings, DAP may be valuable as an anti-biofilm agent.

There are two major steps in *P. aeruginosa* biofilm development (a) cell surface attachment and (b) cell-cell interactions (Qu et al., 2016) (Qu et al., 2016). Bacterial adhesion is the initial stage of biofilm formation, and inhibiting bacterial adhesion is important to control bacterial biofilms (Rasamiravaka et al., 2015). Different types of motilities play unique roles in the surface colonization and biofilm formation of *P. aeruginosa* (Pejcic et al., 2020). Adhesion of *P. aeruginosa* is closely related to flagella, pili, and their swimming, swarming, and twitching motilities; of these, the twitching movement is an important step for bacteria to form micro-colonies and biofilms (Kuhn et al., 2021). Recent studies have reported that some compounds derived from plants can inhibit *P. aeruginosa* motility (O’may and Tufenkji, 2011; Luo et al., 2017; Topa et al., 2018). In this study, our results showed that DAP can effectively inhibit the swimming, swarming, and twitching mobility of *P. aeruginosa*, indicating that DAP can influence the functions of flagella and Type IV pili and facilitate the inhibition of bacterial adhesion, colonization, and biofilm formation.

Pyocyanin produced by *P. aeruginosa* also plays an important role in infection. Pyocyanin is typically produced when bacterial density is high (Folkesson et al., 2012). Recent studies have demonstrated that pyocyanin has several physiological functions that help bacteria evade the human immune system. For example, the production of reactive oxygen free radicals triggering oxidative stress in host cells was found to be mediated by pyocyanin, thereby playing the role of killer cells (Behzadi et al., 2021). Pyocyanin can act as a signaling molecule to regulate the QS system (Dietrich et al., 2006). The development of collective antibiotic tolerance was mediated by pyocyanin (Zhu et al., 2019). In this experiment, pyocyanin production at various DAP concentrations was detected. The results showed that pyocyanin production decreased with increasing DAP concentration, indicating that DAP can inhibit pyocyanin production.

The formation of biofilm is a complex process, which mainly includes the following stages: adsorption, adhesion, formation of microcolonies, maturation, and dispersal (Guzman-Soto et al., 2021). Compared with planktonic bacteria, *P. aeruginosa* biofilms have stronger antibiotic tolerance and are more resistant to host responses (Cendra and Torrents, 2021). However, most antibiotic research currently address the treatment of acute infections caused by planktonic bacteria since few commercial antibiofilm drugs are available (Yu and Chua, 2020). Natural products have recently attracted increased attention due to their safety and antibiofilm effects. Recent studies have shown that gingerols, proanthocyanins, and condensed tannins, which are extracted from Ginger (Zingiber Officinale) and wild blueberry (Vaccinium Angustifolium), have antibiofilm effects (Miari et al., 2020). Topa et al. showed that Cinnamaldehyde could disrupt biofilm formation and the swarming motility of *P. aeruginosa* (Topa et al., 2018). DAP is a natural compound produced by various plants. Studies have shown that DAP has several biological activities, which include antibacterial, antifungal, anticoagulant, antioxidant, anticancer, and anti-inflammatory (Cespedes et al., 2006; Yang et al., 2016). However, no studies have shown that DAP can prevent *P. aeruginosa* biofilm formation or eradicate formed biofilm. In this study, crystal violet staining, CFU enumeration assay, and SEM were used to determine whether DAP could inhibit biofilm formation and eradicate biofilm. Our quantitative assay results and morphological observations (SEM) indicated that DAP produced inhibitory effects on the *P. aeruginosa* biofilm at concentrations of 1.781, 0.890, and 0.445 mg/mL. However, DAP at 1.781 mg/mL had an inhibitory effect on the growth of *P. aeruginosa*. DAP at concentrations of 0.890 and 0.445 mg/ml inhibited the biofilm formation of *P. aeruginosa* without affecting bacterial growth. The effects of DAP on *P. aeruginosa* growth and biofilm in this study are consistent with those reported by a previous study (Kumar et al., 2020). When the drug concentration could not inhibit bacterial growth, DAP acted as an anti-biofilm agent; at higher levels, it acted on multiple cellular targets and showed nonspecific broad-spectrum antimicrobial effects. Our study also found that DAP eradicated *P. aeruginosa* biofilms at concentrations of 1.781 and 0.890 mg/mL.

Traditional Chinese Medicine (TCM), including herbal medicine, acupuncture, and moxibustion, has been continuously developed in Chinese history (Tang et al., 2008). TCM involves unique therapeutic approaches to preventing and treating several diseases. Furthermore, TCM has a long history and rich experience in curing infectious diseases (Pang and Zhu, 2021). Chinese herbal medicine from TCM has the advantages of fewer side effects, effective inhibition of pathogens, and promotion of host immunity (Yu et al., 2017; Ma et al., 2019). As a traditional Chinese medicine, DAP exerts inhibitory effect against a variety of bacterial pathogens. Various natural product extracts from traditional Chinese medicine demonstrate antibacterial and antibiofilm activities against *P. aeruginosa*; these included tea polyphenols, andrographolide, and baicalein (Ma et al., 2012; Yin et al., 2015; Luo et al., 2017). DAP as a safe and non-toxic natural product has been widely studied, and research has shown that it has antimicrobial effects on *Staphylococcus aureus*, *Helicobacter pylori*, and *R. solanacearum* (Zhang et al., 2019; Wang et al., 2019; Yang et al., 2021). However, the antimicrobial and anti-biofilm effects of DAP on *P. aeruginosa* have never been explored. In this study, the potential DAP to inhibit *P. aeruginosa* PAO1 motility and pyocyanin production was investigated, and its antibiofilm activity was assessed using different methods. We showed the ability of DAP to effectively inhibit and eradicate *P. aeruginosa* biofilm, reduce the production of pyocyanin, and inhibit the motility of *P. aeruginosa*.

In conclusion, our study is the first to investigate the antibacterial and anti-biofilm effects of DAP against *P. aeruginosa*. The results show that DAP inhibited *in vitro* production of pyocyanin, bacterial motility, and biofilm formation. DAP demonstrated antimicrobial effects against *P. aeruginosa* at high concentrations and anti-biofilm effects at concentrations that do not inhibit bacterial growth. Furthermore, the QS system plays an irreplaceable role in regulating *P. aeruginosa* biofilm formation, the release of virulence factors, and bacterial motility. Nevertheless, the mechanism underlying the anti-QS system of DAP was not explored, which is the limitation of the study. Further studies should investigate this mechanism. DAP can be used as a novel anti-biofilm and antibacterial compound against *P. aeruginosa*. However, further research is needed to clarify the pharmacokinetic and pharmacodynamic characteristics of DAP in animals infected with *P. aeruginosa* biofilms before it can enter clinical trials.

## Data availability statement

The raw data supporting the conclusions of this article will be made available by the authors, without undue reservation.

## Author contributions

Conceptualization: KW QJW. Data curation: KW QJW. Formal analysis: ZJY LMY DBL SSL. Investigation: ZJY LMY DBL SSL WSD LZ JHL JLL. Methodology: ZJY LMY DBL SSL WSD LZ JHL JLL. Project administration: KW QJW. Resources: ZJY LMY DBL SSL. Software: ZJY LMY DBL SSL. Supervision: KW QJW. Validation: KW QJW. Visualization: ZJY DBL KW QJW. Writing – original draft: ZJY DBL KW QJW. Writing – review & editing: ZJY DBL KW QJW. All authors contributed to the article and approved the submitted version.

## Funding

The work was supported by National Natural Scientific Funds (82260023), Guangxi Natural Scientific Funds (2022GXNSFAA035646), Guangxi Key R&D Program (GuiKe AB22035014), Advanced Innovation Teams and Xinghu Scholars Program of Guangxi Medical University and Collaborative Innovation Centre of Regenerative Medicine and Medical Bioresource Development and Application Co-constructed by the Province and Ministry.

## Acknowledgments

I would like to express my gratitude to Yingchuan Zhou, Yuding Jiang, and Jing Luo. They have helped me a lot in my studies.

## Conflict of interest

The authors declare that the research was conducted in the absence of any commercial or financial relationships that could be construed as a potential conflict of interest.

## Publisher’s note

All claims expressed in this article are solely those of the authors and do not necessarily represent those of their affiliated organizations, or those of the publisher, the editors and the reviewers. Any product that may be evaluated in this article, or claim that may be made by its manufacturer, is not guaranteed or endorsed by the publisher.
